# Nanoparticle Engineering in Modern Vaccinology: From Delivery Platforms to Immune-Programming Architectures

**DOI:** 10.3390/molecules31142501

**Published:** 2026-07-17

**Authors:** Andrey Bogoyavlenskiy, Vladimir Berezin, Madina Alexyuk, Pavel Alexyuk, Elmira Omirtayeva

**Affiliations:** Research and Production Center for Microbiology and Virology, Almaty 050010, Kazakhstan; vberezin359@gmail.com (V.B.);

**Keywords:** nanovaccines, lipid nanoparticles, virus-like particles (VLPs), ISCOMs, adjuvants, antigen display, immunoengineering

## Abstract

Recent advances in vaccinology have accelerated the shift from conventional live-attenuated and inactivated vaccines toward subunit and nucleic acid-based platforms. Although these next-generation vaccines offer improved safety, rapid adaptability, and manufacturing flexibility, their relatively low intrinsic immunogenicity often requires efficient adjuvants and delivery systems. Nanoparticle-based vaccine platforms have therefore emerged as versatile tools capable of protecting antigens, improving targeted delivery, and modulating both innate and adaptive immune responses. This review summarizes the major classes of nanovaccine platforms, including lipid and polymeric nanoparticles, self-assembling protein nanostructures such as virus-like particles and ferritin nanocages, saponin-based self-assembling complexes, and inorganic nanomaterials. Particular attention is given to how vaccine performance is determined not only by material composition but also by nanoparticle physicochemical properties, biodistribution, cellular uptake, and mechanisms of immune activation. We further discuss the major challenges limiting clinical translation, including scalable manufacturing, safety evaluation, quality control, regulatory requirements, and long-term biocompatibility. Finally, emerging strategies involving hybrid and personalized nanovaccine platforms are highlighted, illustrating how nanotechnology and immunoengineering are transforming vaccine development for both prophylactic and therapeutic applications.

## 1. Introduction

Vaccination remains one of the most effective public health interventions for the prevention of infectious diseases and has dramatically reduced the global burden of morbidity and mortality [1,2,3]. Nevertheless, despite the remarkable success of conventional vaccines, important challenges remain, including limited efficacy against highly variable pathogens, inadequate induction of cellular and mucosal immunity, poor stability of certain antigens, and the need for safe and efficient delivery systems [4,5]. These limitations have driven the rapid development of nanotechnology-based vaccine platforms capable of integrating antigen delivery with programmable immune modulation [6,7]. However, conventional vaccine technologies continue to face important limitations, including poor stability of labile antigens, inefficient delivery to antigen-presenting cells (APCs), rapid antigen clearance, limited induction of cellular immunity, and insufficient immunogenicity against highly variable or intracellular pathogens [8]. These challenges have stimulated the development of advanced delivery systems capable of improving both antigen presentation and immune regulation.

Nanotechnology has emerged as one of the most transformative approaches in modern vaccinology by enabling the rational design of delivery systems that actively participate in immune modulation rather than merely serving as passive antigen carriers [6]. Owing to their tunable physicochemical properties, large surface-area-to-volume ratio, and versatile functionalization strategies, nanoparticles can protect antigens from premature degradation, facilitate targeted delivery to lymphoid tissues, promote intracellular trafficking, and enable the co-delivery of immunostimulatory molecules. As a result, nanoplatforms provide opportunities not only to enhance vaccine potency but also to direct the magnitude, quality, and durability of immune responses [9,10].

A defining feature of contemporary nanovaccine design is the ability to engineer particle architecture at multiple structural levels. Parameters such as particle size, morphology, surface charge, elasticity, chemical composition, degradation kinetics, and surface functionalization collectively determine the biological identity of nanoparticles following administration [11,12]. These characteristics influence biodistribution, protein corona formation, lymphatic transport, cellular uptake, intracellular processing, antigen presentation, and activation of innate immune signaling pathways. Consequently, the immunological performance of nanovaccines is governed not by material composition alone but by the integrated interaction between physicochemical design and biological processes.

The concept of nanoparticle engineering has therefore shifted from empirical formulation toward mechanism-driven design. Rather than optimizing individual physicochemical parameters independently, current strategies seek to integrate multiple design elements that collectively regulate antigen persistence, intracellular delivery, immune cell targeting, and spatiotemporal control of immune activation. This systems-oriented approach has considerably expanded the range of vaccine platforms available for both prophylactic and therapeutic applications, including infectious diseases, cancer immunotherapy, and personalized medicine.

The principal engineering parameters that govern nanoparticle vaccine performance and their major biological consequences are summarized in Table 1.

Collectively, these design parameters establish a common engineering framework that is applicable across virtually all nanoparticle-based vaccine platforms. Although individual nanocarriers differ substantially in material composition and fabrication strategies, they share common biological objectives: preserving antigen integrity, promoting efficient delivery to immune compartments, and directing adaptive immune responses through controlled activation of innate immunity [32].

**Figure 1:**
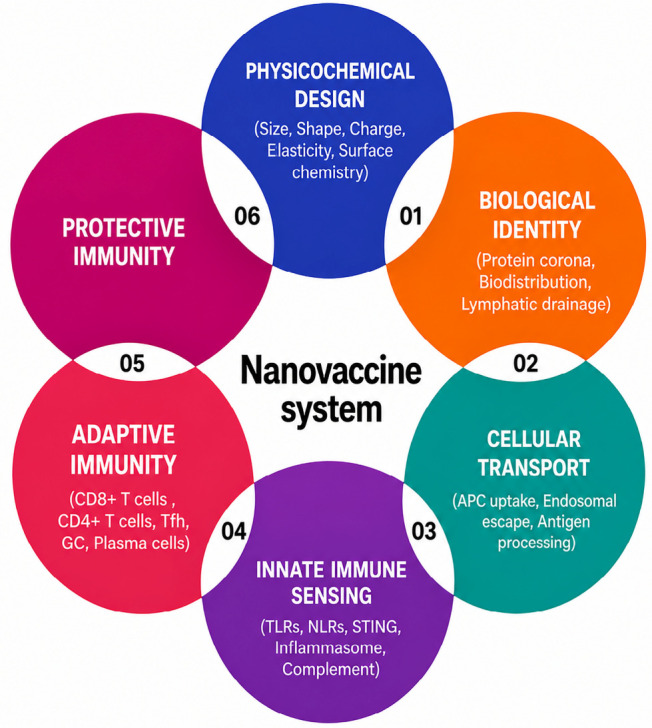
Unified mechanistic framework governing nanoparticle vaccine performance.

Accordingly, nanovaccine systems can be conceptualized as integrated immunoengineering platforms in which vaccine performance is determined by the coordinated interaction of the following parameters/processes: physicochemical design, biological identity, cellular transport, innate immune sensing, adaptive immunity, protective immunity (Figure 1).

Although these engineering principles are universal, they are implemented differently by individual nanocarrier platforms. Lipid nanoparticles, polymeric nanoparticles, nanoemulsions, extracellular vesicles, virus-like particles, and inorganic nanomaterials each employ distinct structural strategies to achieve similar immunological objectives [33,34,35,36,37,38,39]. The following sections therefore examine the mechanistic characteristics, biological behavior, advantages, limitations, and translational potential of the major nanoparticle platforms currently used in vaccine development within this unified conceptual framework.

## 2. Classification of Nanocomplex Platforms for Vaccine Engineering

Modern nanovaccine systems represent functionally diverse yet conceptually unified platforms aimed at efficient antigen delivery and/or immunomodulatory activation of innate and adaptive immune responses [33,34,35,36,37]. Depending on material composition, self-assembly principles, and immunobiological function, these systems can be broadly classified into four major categories (Table 2, Table 3 and Table 4).

### 2.1. Lipid and Polymeric Nanoparticle Systems

Lipid-, polymer-, nanoemulsion-, and biomimetic vesicular systems represent the principal classes of engineered and biologically derived nanocarriers used in contemporary vaccinology [38,39,40,58,59]. Although these platforms differ substantially in their chemical composition, self-assembly mechanisms, and structural organization, they share a common objective: to protect antigenic cargo from premature degradation, ensure its spatiotemporally controlled delivery to immune compartments, and actively modulate both innate and adaptive immune responses [59,60,61]. Rather than acting as passive delivery vehicles, modern nanocarriers function as programmable immunological platforms whose physicochemical architecture determines the magnitude, quality, and durability of vaccine-induced immunity [62,63].

#### 2.1.1. Physicochemical Architecture and Antigen Presentation

Beyond material composition, nanoparticle size, morphology, elasticity, surface charge, and hydrophilicity critically determine their biological behavior following administration [15,16,18,64]. These physicochemical characteristics influence lymphatic drainage, cellular uptake, intracellular trafficking, and antigen persistence, thereby shaping both innate immune activation and subsequent adaptive immune responses [25,65]. Consequently, nanoparticle engineering increasingly focuses on optimizing physicochemical architecture rather than simply selecting carrier materials.

Immediately after administration, nanoparticles encounter biological fluids where they become coated with plasma proteins, forming a dynamic protein corona. This adsorbed protein layer effectively replaces the original nanoparticle surface as the interface recognized by immune cells. Protein corona composition influences complement activation, opsonization, circulation time, lymphatic transport, and cellular uptake, making it one of the major determinants of nanoparticle biodistribution and immunogenicity [66,67].

Lipid nanoparticles (LNPs) constitute the most clinically advanced platform for nucleic acid delivery. They are formed through the self-assembly of ionizable lipids with helper phospholipids, cholesterol, and PEGylated lipids into highly organized lipid–nucleic acid complexes capable of encapsulating mRNA, siRNA, or plasmid DNA with high efficiency [68]. Their defining mechanistic feature is pH-dependent ionization: ionizable lipids become positively charged under acidic conditions, facilitating nucleic acid encapsulation during formulation and promoting endosomal membrane destabilization following cellular uptake, while remaining largely neutral at physiological pH to minimize nonspecific protein adsorption and systemic toxicity.

Polymeric nanoparticles are typically fabricated from biodegradable polymers including PLGA, PLA, PGA, polycaprolactone, and chitosan [69,70]. Antigens may either be encapsulated within the polymeric matrix or adsorbed onto the particle surface, enabling precise control over antigen localization and release kinetics [71,72,73]. Polymer molecular weight, copolymer composition, crystallinity, and hydrophilicity collectively regulate hydrolytic degradation, allowing antigen release profiles ranging from rapid burst release to sustained depot formation extending over weeks or months [74,75].

Nanoemulsions consist of kinetically stabilized oil-in-water or water-in-oil droplets whose antigen presentation relies primarily on interfacial adsorption rather than encapsulation within a solid matrix [76]. Their large interfacial surface area facilitates incorporation of lipophilic antigens and adjuvants while promoting intimate interactions with cellular membranes and enhancing local immune activation.

Biologically derived extracellular vesicles, including exosomes, and biomimetic membrane-coated nanoparticles represent an emerging class of delivery systems that exploit naturally evolved mechanisms of intercellular communication [77]. Their membrane composition preserves endogenous lipids, membrane proteins, adhesion molecules, and nucleic acids inherited from parental cells, thereby facilitating receptor-mediated targeting and improving biocompatibility. However, biological origin also introduces substantial heterogeneity in composition, manufacturing reproducibility, and functional activity compared with synthetic nanocarriers.

#### 2.1.2. Biological Identity, Lymphatic Trafficking, and Cellular Transport

Following administration, nanoparticle transport is governed not only by particle size but also by their dynamic biological identity resulting from protein corona formation [17,65]. Nanoparticles ranging from approximately 20 to 100 nm exhibit the highest efficiency of passive lymphatic drainage, whereas larger particles rely increasingly on active transport by migratory antigen-presenting cells [25].

Following entry into lymphatic vessels, nanoparticles accumulate within draining lymph nodes where they encounter dendritic cells, macrophages, and B lymphocytes. Cellular internalization proceeds through multiple endocytic pathways—including clathrin-mediated endocytosis, caveolin-dependent endocytosis, macropinocytosis, and phagocytosis—with the relative contribution of each pathway determined by particle size, elasticity, geometry, and surface chemistry [21,78].

Among current delivery systems, LNPs possess the greatest capacity for cytosolic delivery owing to efficient endosomal escape mediated by pH-dependent membrane destabilization induced by ionizable lipids. This property enables intracellular translation of mRNA vaccines or cytosolic delivery of RNA therapeutics and represents one of the principal reasons for the clinical success of LNP-based vaccines [38,39,79,80].

By contrast, polymeric nanoparticles generally undergo slower intracellular processing, releasing antigen gradually as biodegradable polymers hydrolyze within endosomal and lysosomal compartments. Although this prolonged antigen persistence enhances sustained immune stimulation, efficient endosomal escape often requires additional engineering strategies, including proton-sponge polymers, fusogenic peptides, or membrane-disruptive lipids [69,70,81,82,83].

Nanoemulsions display a distinct depot-based transport mechanism characterized by prolonged antigen retention at the injection site. This localized inflammatory microenvironment promotes continuous recruitment of monocytes, neutrophils, and dendritic cell precursors, resulting in sustained antigen transport to draining lymph nodes over extended periods [84,85,86].

Extracellular vesicles exhibit highly specialized biodistribution mediated by endogenous membrane proteins, integrins, and tetraspanins that facilitate receptor-dependent cellular targeting. Their endogenous origin reduces recognition by phagocytic clearance systems while supporting long-distance intercellular communication and, in certain experimental models, transport across physiological barriers such as the blood–brain barrier [87,88,89,90].

#### 2.1.3. Innate Immune Sensing

Nanoparticle-induced innate immune activation is governed by both material composition and physicochemical architecture. In addition to determining antigen delivery, nanoparticles actively regulate the activation of pattern-recognition receptors, inflammasomes, complement pathways, and inflammatory signaling cascades [40,62,91].

LNPs activate innate immunity primarily through intracellular sensing of delivered nucleic acids by endosomal Toll-like receptors (TLR3, TLR7, and TLR8), cytosolic RIG-I-like receptors, and additional RNA-sensing pathways. Furthermore, ionizable lipids themselves may induce membrane stress responses, inflammasome activation, and transient cytokine production that collectively contribute to vaccine reactogenicity while simultaneously enhancing adaptive immune priming [38,39,80,92,93,94].

Polymeric nanoparticles generally exhibit moderate intrinsic adjuvant activity resulting from enhanced phagocytosis and activation of inflammasome-associated pathways. Their degradation products, including lactic and glycolic acid released from PLGA matrices, modify local tissue pH and influence recruitment, activation, and metabolic programming of innate immune cells [69,70,95,96].

Nanoemulsions represent among the strongest inducers of local inflammatory responses. Membrane perturbation induced by surfactants and lipid interfaces stimulates robust production of chemokines and pro-inflammatory cytokines, leading to rapid recruitment of antigen-presenting cells and efficient amplification of antigen presentation within draining lymph nodes [84,86].

Exosomes typically exhibit low basal immunogenicity because their endogenous membrane composition minimizes activation of pattern-recognition receptors and complement pathways. Nevertheless, genetic engineering, membrane functionalization, or loading with immunostimulatory molecules enables precise modulation of their immunological properties while preserving excellent biocompatibility [87,88,97].

Surface physicochemistry additionally regulates complement activation. Controlled complement deposition may enhance antigen-presenting cell recruitment and vaccine efficacy, whereas excessive complement activation has been associated with complement activation-related pseudoallergy (CARPA), particularly for certain lipid-based formulations [13,31,98].

#### 2.1.4. Adaptive Immune Programming

Beyond innate immune activation, nanoparticle architecture profoundly influences adaptive immune differentiation by regulating antigen persistence, intracellular trafficking, and presentation through major histocompatibility complex pathways.

Efficient cytosolic antigen delivery promotes cross-presentation via MHC class I molecules, leading to robust priming of cytotoxic CD8^+^ T lymphocytes that are essential for antiviral immunity and therapeutic cancer vaccination. Conversely, prolonged extracellular antigen availability favors MHC class II presentation, differentiation of T follicular helper cells, germinal center formation, affinity maturation of B cells, and development of long-lived plasma cells capable of sustained antibody production.

The duration of antigen exposure also influences immunological memory. Controlled-release polymeric nanoparticles frequently generate prolonged germinal center reactions, whereas LNP-mediated transient antigen expression elicits rapid but potent immune activation. These complementary immune programming strategies provide opportunities for tailoring vaccine responses according to pathogen biology and desired clinical outcomes.

#### 2.1.5. Translational Considerations and Comparative Perspective

Among currently available nanocarriers, LNPs possess the highest degree of clinical validation owing to their successful application in mRNA vaccines. Their principal limitations include preferential hepatic accumulation, anti-PEG immune responses following repeated administration, and formulation-specific cold-chain requirements. Nevertheless, mature manufacturing processes and high encapsulation efficiency establish LNPs as the current benchmark for nucleic acid vaccine delivery [38,80,99,100].

Polymeric nanoparticles provide exceptional flexibility for controlled antigen release and co-delivery of immunomodulators but continue to face challenges related to manufacturing reproducibility, degradation variability, and scale-up [58,69,72].

Nanoemulsions remain among the most successful clinically approved adjuvant systems because of their strong immunostimulatory properties and compatibility with both systemic and mucosal vaccination strategies. However, oxidative instability and stringent manufacturing requirements remain important considerations [101,102,103].

Extracellular vesicles and biomimetic membrane-coated nanoparticles represent one of the fastest-growing areas of vaccine nanotechnology. Their exceptional targeting capabilities and biocompatibility are balanced by persistent challenges involving scalable production, purification standardization, batch heterogeneity, and regulatory classification [87,88,104].

#### 2.1.6. Summary

Collectively, lipid nanoparticles, polymeric nanoparticles, nanoemulsions, and biomimetic vesicular systems represent complementary yet mechanistically distinct strategies for vaccine delivery. Despite their diverse origins and structural organization, their biological performance is governed by a common framework integrating physicochemical architecture, biological identity, lymphatic transport, innate immune sensing, and adaptive immune programming. Future nanoparticle vaccines will increasingly rely on the rational integration of these engineering principles, transforming nanocarriers from passive delivery vehicles into programmable immunological platforms capable of directing highly specific and durable protective immunity.

### 2.2. Saponin-Based Self-Assembling Nanoplatforms

Saponin-based nanoplatforms constitute a distinct class of self-assembling immunostimulatory systems in which structural organization, antigen delivery, and adjuvant activity are intrinsically integrated within a single nanoscale architecture [105,106,107]. Unlike conventional nanoparticle carriers that primarily function as delivery vehicles and often require separate immunostimulatory adjuvants, saponin-based nanocomplexes combine both functions within the same supramolecular structure [107,108]. This unique property enables simultaneous protection of antigens, efficient delivery to antigen-presenting cells (APCs), and potent activation of innate and adaptive immune responses [109,110]. Classical immunostimulating complexes (ISCOMs), ISCOMATRIX formulations, and the clinically approved Matrix-M adjuvant represent the best-established examples of this strategy; however, recent studies demonstrate that numerous plant-derived saponins possess similar self-assembling properties, considerably expanding the diversity of vaccine nanoplatforms [44,111,112,113].

#### 2.2.1. Structural Organization and Self-Assembly

The ability of saponins to form nanoscale supramolecular assemblies arises from their amphiphilic molecular architecture, consisting of a hydrophobic triterpenoid or steroidal aglycone linked to one or more hydrophilic oligosaccharide chains [106,114]. In the presence of cholesterol and phospholipids, these molecules spontaneously self-assemble into highly ordered nanoparticles stabilized by hydrophobic interactions, hydrogen bonding, and sterol–saponin complex formation [115,116]. Depending on molecular structure and formulation conditions, saponins may generate cage-like ISCOM particles, vesicular nanostructures, micellar assemblies, or hybrid lipid nanoparticles.

Classical ISCOMs are formed from cholesterol, phospholipids, and Quil A extracted from Quillaja Saponaria. During particle assembly, vaccine antigens are incorporated directly into the nanostructure, allowing simultaneous antigen stabilization and multivalent presentation. In contrast, ISCOMATRIX and Matrix-M primarily function as nanoparticulate adjuvants that are administered together with soluble antigens, thereby providing greater flexibility for vaccine formulation while preserving the immunostimulatory properties of saponins [44,111].

Although Quillaja saponaria remains the principal commercial source of vaccine-grade saponins, increasing evidence indicates that amphiphilic saponins isolated from numerous other plant species can also participate in the formation of stable immunostimulatory nanostructures. Saponins obtained from Panax ginseng, Astragalus membranaceus, Glycyrrhiza glabra, Platycodon grandiflorus, Camellia oleifera, Aesculus hippocastanum, and several Sapindus and Pulsatilla species have demonstrated the ability to associate with cholesterol-containing lipid membranes or form self-assembled nanoparticles exhibiting adjuvant activity [46,47,48,117,118]. Although many of these systems remain at the experimental stage, they frequently display lower hemolytic activity and improved biocompatibility compared with Quil A-derived formulations, making them attractive candidates for next-generation vaccine delivery systems.

These findings have shifted current research toward understanding the structure–activity relationships governing saponin self-assembly, membrane interactions, and immune activation. Rational selection of individual saponin fractions or chemical modification of their carbohydrate moieties is increasingly being explored to optimize nanoparticle stability, reduce reactogenicity, and improve immunological performance.

#### 2.2.2. Biological Transport and Antigen Presentation

Following administration, saponin nanocomplexes efficiently drain through afferent lymphatic vessels owing to their nanoscale dimensions and preferentially accumulate within draining lymph nodes. There they are rapidly internalized by dendritic cells, macrophages, and other professional APCs through multiple endocytic pathways. Besides facilitating antigen transport, nanoparticle architecture promotes prolonged antigen retention within lymphoid tissues, thereby increasing the probability of productive interactions with immune cells [44,119].

A defining feature of saponin-based nanoplatforms is their exceptional ability to promote cross-presentation of exogenous antigens through major histocompatibility complex class I (MHC-I) molecules. Interactions between saponins and cholesterol-rich endosomal membranes facilitate antigen translocation into the cytosol, thereby enhancing activation of cross-presenting dendritic cells and stimulating robust CD8^+^ cytotoxic T-cell responses [110]. Simultaneously, efficient processing through MHC-II pathways supports activation of CD4^+^ T-helper cells, germinal center formation, T follicular helper cell differentiation, and durable antibody production [120]. Consequently, these platforms effectively induce both humoral and cellular immunity, a characteristic that distinguishes them from many conventional adjuvant systems.

Several experimental studies further indicate that intranasal administration of saponin nanocomplexes promotes efficient antigen delivery to mucosal lymphoid tissues and stimulates secretory IgA production, highlighting their considerable potential for mucosal vaccine development [106].

#### 2.2.3. Innate Immune Activation

The potent immunological activity of saponin nanocomplexes derives primarily from the intrinsic biological properties of triterpenoid saponins combined with their supramolecular organization. Following interaction with cholesterol-rich plasma and endosomal membranes, saponins induce transient membrane remodeling that enhances antigen uptake and intracellular trafficking while simultaneously activating dendritic cells [120,121].

Cellular internalization is accompanied by upregulation of co-stimulatory molecules, increased production of inflammatory cytokines and chemokines, and activation of multiple innate immune signaling pathways, including inflammasome-associated mechanisms and lysosomal stress responses. These events collectively amplify antigen presentation and support efficient priming of adaptive immunity [110].

Unlike many conventional adjuvants that predominantly favor either Th1- or Th2-polarized responses, optimized saponin formulations generally promote a balanced immune profile characterized by strong antibody responses, efficient cytotoxic T-cell activation, and sustained immunological memory. The magnitude and quality of these responses depend on the molecular structure of individual saponins, nanoparticle composition, and formulation strategy, emphasizing the importance of rational nanoparticle engineering [122].

#### 2.2.4. Translational Perspective

Among currently available saponin-based nanoplatforms, Matrix-M represents the most clinically advanced example of rational saponin engineering. Composed of highly purified fractions of Quillaja saponaria saponins formulated with cholesterol and phospholipids, Matrix-M has demonstrated substantial enhancement of antigen-presenting cell recruitment, lymph node activation, cytokine production, and adaptive immune responses in both preclinical and clinical studies. Its successful incorporation into licensed human vaccines has established proof of concept for self-assembling saponin nanoparticles as clinically applicable vaccine adjuvants [112,123].

Current research is extending this concept beyond Quillaja-derived formulations by investigating alternative plant saponins, semisynthetic derivatives, and multifunctional hybrid nanocomplexes designed to combine antigen delivery, immune modulation, and tissue-specific targeting within a single platform. These developments are expected to broaden the applicability of saponin-based nanotechnologies for prophylactic vaccines, therapeutic cancer vaccines, mucosal immunization, and personalized immunotherapy [124].

#### 2.2.5. Summary

Collectively, saponin-based self-assembling nanoplatforms represent a unique class of vaccine delivery systems in which supramolecular organization and intrinsic immunostimulatory activity are inseparably linked. Continued exploration of structurally diverse plant-derived saponins, together with advances in nanoparticle engineering and formulation science, is transforming these naturally occurring amphiphiles from traditional adjuvants into versatile immunoengineering platforms capable of directing antigen delivery, innate immune activation, and adaptive immune programming with high precision.

### 2.3. Self-Assembling Protein Nanoplatforms: Virus-like Particles and Ferritin Nanocages

Self-assembling protein nanoplatforms have emerged as one of the most successful strategies in modern vaccine engineering by exploiting the intrinsic ability of proteins to spontaneously assemble into highly ordered nanostructures. Among these platforms, virus-like particles (VLPs) and ferritin nanocages are the most extensively investigated and clinically advanced systems. Despite their different biological origins, both platforms share a common design principle: the precise organization of multiple antigen copies within a defined nanoscale architecture that closely mimics the structural characteristics of pathogens while lacking infectious potential. Rather than functioning as conventional delivery vehicles, these protein nanostructures serve as immunological scaffolds that enhance antigen recognition, regulate B-cell activation, and promote coordinated humoral and cellular immune responses [23,125].

#### 2.3.1. Structural Organization and Antigen Display

The exceptional immunogenicity of self-assembling protein nanoparticles arises primarily from their highly ordered structural organization. Both VLPs and ferritin nanocages are formed through spontaneous assembly of multiple protein subunits into symmetrical nanoparticles that display antigens with precise spatial orientation and controlled valency [23,125].

VLPs are generated from viral structural proteins that self-assemble into spherical, icosahedral, or filamentous particles ranging from approximately 20 to 200 nm in diameter. Although devoid of viral genetic material, they faithfully reproduce the external architecture of native virions, preserving conformational epitopes and repetitive antigen organization that efficiently engage the adaptive immune system [125].

Ferritin nanocages originate from the endogenous iron-storage protein ferritin, whose 24 identical or homologous subunits spontaneously assemble into highly symmetrical hollow nanoparticles approximately 12 nm in diameter. Unlike VLPs, ferritin does not inherently resemble viral particles; instead, it provides an exceptionally stable protein scaffold onto which heterologous antigens can be genetically fused or chemically conjugated without disrupting nanoparticle assembly. This modular architecture allows precise control over antigen density, orientation, and molecular spacing [23].

Recent advances in protein engineering have substantially expanded the versatility of both platforms. Site-specific conjugation technologies, including SpyTag/SpyCatcher systems, sortase-mediated ligation, click chemistry, and streptavidin–biotin coupling, enable highly controlled antigen decoration while preserving native protein conformation. Furthermore, computationally designed self-assembling nanoparticles and mosaic antigen-display systems now permit simultaneous presentation of multiple antigenic variants, broadening immune recognition and increasing protection against genetically diverse pathogens [126].

#### 2.3.2. Immune Recognition and Biological Behavior

The nanoscale dimensions and highly repetitive antigen arrangement characteristic of protein nanoplatforms profoundly influence immune recognition. Following administration, VLPs and ferritin nanoparticles efficiently drain into regional lymph nodes, where they interact directly with dendritic cells, macrophages, follicular dendritic cells, and B lymphocytes. A defining advantage of these platforms is the repetitive surface presentation of antigens, which efficiently cross-links B-cell receptors and lowers the activation threshold for naïve B cells. This multivalent interaction promotes rapid germinal center formation, differentiation of T follicular helper cells, affinity maturation, and generation of long-lived plasma cells capable of sustained antibody production [127,128].

Internalized antigens are processed predominantly through MHC class II pathways, supporting robust CD4^+^ T-helper cell activation. Depending on nanoparticle composition, intracellular trafficking, and vaccine formulation, cross-presentation through MHC class I pathways may also occur, resulting in efficient priming of cytotoxic CD8^+^ T lymphocytes. Consequently, self-assembling protein nanoparticles provide a balanced platform capable of inducing both potent neutralizing antibody responses and cellular immunity [129].

#### 2.3.3. Innate Immune Activation

Although VLPs and ferritin nanocages share similar structural organization, their interactions with the innate immune system differ substantially. Because VLPs retain many structural characteristics of authentic viruses, they are efficiently recognized by antigen-presenting cells and frequently activate pattern-recognition receptors through encapsulated nucleic acids or viral structural motifs. Depending on the production system and particle composition, VLPs may stimulate Toll-like receptor signaling, type I interferon production, inflammasome activation, and maturation of dendritic cells, thereby providing substantial intrinsic adjuvant activity even in the absence of additional immunostimulants [130,131].

In contrast, ferritin nanocages are derived from a highly conserved endogenous protein and therefore exhibit relatively limited intrinsic immunogenicity. Their excellent biocompatibility minimizes undesired anti-carrier immune responses during repeated immunization, but efficient activation of innate immunity often requires co-formulation with molecular adjuvants or incorporation into multifunctional nanoplatforms. This characteristic provides considerable flexibility for tailoring immune responses according to specific vaccine applications [42,132].

#### 2.3.4. Translational Perspective

Virus-like particles represent one of the most mature nanoparticle technologies in clinical vaccinology and have already been successfully implemented in licensed vaccines against hepatitis B virus, human papillomavirus, hepatitis E virus, and several veterinary pathogens. Their ability to reproduce native viral architecture while eliminating infectious potential has established VLPs as a benchmark platform for prophylactic vaccine development [133].

Ferritin nanocages represent a newer generation of protein nanoscaffolds that have demonstrated remarkable versatility in vaccines targeting influenza viruses, SARS-CoV-2, respiratory syncytial virus, HIV, Ebola virus, coronaviruses, and multiple emerging pathogens. Their structural stability, reproducible self-assembly, and compatibility with computational protein engineering have positioned ferritin among the most promising scaffolds for universal and broadly protective vaccines [134,135].

Current research increasingly integrates computational antigen design, artificial intelligence-guided protein engineering, and de novo designed self-assembling protein nanoparticles to optimize antigen geometry at atomic resolution. Hybrid systems combining ferritin scaffolds, virus-like particle architecture, and modular antigen assembly technologies are expected to further improve antigen presentation, broaden immune coverage, and facilitate rapid development of vaccines against newly emerging infectious diseases [136,137].

#### 2.3.5. Summary

Collectively, virus-like particles and ferritin nanocages exemplify the transition from conventional antigen delivery toward structurally programmed vaccine design. By precisely controlling nanoscale architecture, antigen valency, and spatial organization, these self-assembling protein nanoplatforms efficiently coordinate B-cell activation, T-cell priming, and long-term immunological memory (Table 5). Continued advances in computational protein engineering and modular nanoparticle assembly are expected to further expand their role as versatile platforms for next-generation prophylactic, therapeutic, and universal vaccines.

### 2.4. Inorganic Nanoplatforms for Vaccine Delivery and Immunotherapy

Inorganic nanomaterials constitute a unique class of engineered vaccine platforms whose biological behavior is governed not only by nanoscale dimensions and surface chemistry but also by intrinsic physicochemical properties that are absent in biological nanocarriers. Optical plasmon resonance, magnetic responsiveness, catalytic activity, electrical conductivity, fluorescence, and exceptional structural stability enable these materials to perform functions extending far beyond conventional antigen delivery [34,49]. Consequently, inorganic nanoparticles are increasingly being developed as multifunctional immunoengineering platforms that integrate vaccine delivery, immune modulation, diagnostic imaging, and externally controlled therapeutic activation.

Unlike lipid-, polymer-, and protein-based nanoparticles, which rely primarily on biological self-assembly and biodegradation, inorganic nanomaterials are synthesized with precise control over particle size, morphology, crystallinity, porosity, and surface functionalization [138,139]. This high degree of structural control enables rational optimization of antigen loading, intracellular trafficking, biodistribution, and interactions with immune cells. Moreover, their unique physical properties permit external regulation of nanoparticle behavior using light, magnetic fields, ultrasound, or other physical stimuli, providing unprecedented opportunities for spatiotemporal control of immune responses [140].

#### 2.4.1. Structural Organization and Multifunctionality

The structural diversity of inorganic nanoparticles provides remarkable flexibility for vaccine engineering. Gold nanoparticles (AuNPs), silver nanoparticles (AgNPs), mesoporous silica nanoparticles (MSNs), iron oxide nanoparticles (Fe_3_O_4_ and γ-Fe_2_O_3_), calcium phosphate nanoparticles, carbon-based nanomaterials, quantum dots, and emerging nanozymes each possess distinct physicochemical characteristics that determine their biological performance [141,142,143,144].

Gold nanoparticles remain among the most extensively investigated inorganic vaccine platforms. Their chemically inert core, exceptional colloidal stability, and strong affinity for thiol-containing molecules enable dense and spatially controlled immobilization of peptides, proteins, carbohydrates, nucleic acids, and targeting ligands. Such multivalent antigen presentation closely mimics repetitive pathogen-associated structures and enhances B-cell receptor clustering, thereby promoting efficient humoral immune responses. In addition, localized surface plasmon resonance enables photothermal conversion following near-infrared irradiation, creating opportunities for combined vaccination and photothermal immunotherapy [141].

Mesoporous silica nanoparticles differ fundamentally from surface-display platforms by providing a highly ordered porous framework capable of encapsulating large quantities of antigens, nucleic acids, cytokines, and molecular adjuvants. Their tunable pore architecture enables precise control over antigen loading and release kinetics, allowing synchronized delivery of multiple immunologically active components [142].

Magnetic iron oxide nanoparticles introduce an additional functional dimension through their responsiveness to external magnetic fields. Besides serving as antigen carriers, they enable magnetic targeting, magnetic resonance imaging, and localized hyperthermia, thereby combining therapeutic delivery with real-time imaging and externally controlled immune activation [145].

Carbon-based nanomaterials, including graphene oxide, carbon nanotubes, and carbon nanohorns, provide exceptionally large surface areas for biomolecule adsorption and extensive opportunities for chemical functionalization [146]. Their unique electrical and mechanical properties have stimulated growing interest in hybrid vaccine systems integrating antigen delivery with biosensing and immunomodulation.

More recently, calcium phosphate nanoparticles, quantum dots, and catalytic nanozymes have emerged as promising alternatives. Calcium phosphate exhibits excellent biocompatibility and biodegradability, quantum dots enable simultaneous imaging and antigen tracking, whereas nanozymes introduce catalytic functions capable of regulating reactive oxygen species (ROS) and inflammatory signaling within immune cells [147].

#### 2.4.2. Biological Transport and Immune Interactions

Following administration, inorganic nanoparticles rapidly interact with biological fluids, leading to the adsorption of proteins, lipids, and other biomolecules onto their surface. This dynamic biomolecular coating, commonly referred to as the protein corona, establishes the biological identity of nanoparticles and profoundly influences cellular recognition, biodistribution, complement activation, and immune clearance. Consequently, immune cells interact primarily with the corona-coated nanoparticle rather than with the original engineered surface [148].

The transport behavior of inorganic nanoparticles is determined by particle size, morphology, aggregation state, and surface chemistry. Nanoparticles within the optimal size range efficiently enter lymphatic capillaries and accumulate in draining lymph nodes, where they encounter dendritic cells, macrophages, and other professional antigen-presenting cells. Cellular internalization occurs predominantly through clathrin-mediated endocytosis, caveolin-mediated uptake, macropinocytosis, or phagocytosis depending on particle characteristics and cell type [149].

Surface engineering plays a central role in controlling these interactions. Polyethylene glycol (PEG), polysaccharides, phospholipid coatings, antibodies, aptamers, mannose residues, and cell-specific peptides are widely employed to reduce nonspecific protein adsorption, prolong systemic circulation, enhance lymphatic transport, and improve selective targeting of immune cell populations [150].

Among inorganic platforms, magnetic nanoparticles offer a unique opportunity for externally guided biodistribution. Application of magnetic fields enables localized accumulation of nanoparticles within selected tissues or lymphoid organs, increasing antigen concentration at desired sites while minimizing systemic exposure. This capability is particularly attractive for personalized cancer vaccines and image-guided immunotherapy [151].

#### 2.4.3. Immune Modulation Beyond Antigen Delivery

Unlike biodegradable biological nanocarriers that primarily function as antigen delivery systems, inorganic nanoparticles actively modulate the immune microenvironment due to their intrinsic physicochemical properties [152,153].

Gold nanoparticles convert absorbed light into localized heat via plasmonic excitation, inducing immunogenic cell death and promoting the release of tumor-associated antigens and damage-associated molecular patterns (DAMPs). This process enhances dendritic cell activation and broadens antigen availability during therapeutic cancer vaccination [154].

Iron oxide nanoparticles generate localized hyperthermia under alternating magnetic fields, facilitating controlled antigen release, improving immune cell infiltration, and enhancing adaptive immune priming. Simultaneously, their magnetic properties enable non-invasive imaging and precise spatial control of therapeutic activation [155].

Catalytic nanomaterials, commonly referred to as nanozymes, represent an emerging generation of inorganic immunomodulators. By mimicking the activity of natural oxidoreductases, these nanoparticles regulate intracellular ROS levels, influence redox-sensitive signaling pathways, and modulate inflammatory responses [156]. Depending on their catalytic activity, nanozymes may either amplify immune activation or suppress excessive inflammation, providing opportunities for precision regulation of vaccine-induced immunity.

Collectively, these stimulus-responsive mechanisms distinguish inorganic nanoplatforms from conventional vaccine carriers by enabling external and programmable control of immune responses.

#### 2.4.4. Innate Immune Activation

The innate immunological activity of inorganic nanoparticles is highly dependent on their physicochemical characteristics rather than material composition alone. Particle size, crystallinity, surface charge, hydrophobicity, protein corona composition, and surface functionalization collectively determine the magnitude and quality of immune activation [157].

Gold nanoparticles generally exhibit low intrinsic immunogenicity but efficiently enhance antigen presentation following surface conjugation of immunogens or adjuvants. Mesoporous silica nanoparticles have been shown to activate inflammasome-associated signaling pathways and promote dendritic cell maturation under appropriate formulation conditions. Iron oxide nanoparticles influence macrophage polarization, ROS production, and cytokine secretion while simultaneously facilitating antigen presentation through magnetic targeting [158,159].

Silver nanoparticles display pronounced antimicrobial and antiviral activity resulting from oxidative stress, membrane disruption, and sustained release of silver ions. However, these same mechanisms may induce excessive inflammatory responses and cytotoxicity, limiting their translational applicability [160,161].

Carbon-based nanomaterials exhibit highly variable immunological behavior depending on their structural organization and surface chemistry. Functionalized graphene derivatives generally demonstrate improved biocompatibility, whereas poorly dispersed carbon nanotubes may induce chronic inflammation and granulomatous reactions. Consequently, careful control of surface modification remains essential for their safe biomedical application [162,163].

Overall, innate immune activation by inorganic nanoparticles is considerably more tunable—but also less predictable—than that observed for biological nanoplatforms. Rational engineering of surface chemistry therefore represents the principal strategy for balancing immunostimulatory efficacy with biocompatibility.

#### 2.4.5. Translational Perspective

Inorganic nanoparticles have demonstrated substantial potential across numerous areas of vaccinology and immunotherapy. Gold nanoparticles are widely investigated for multivalent antigen display and photothermal cancer vaccination. Mesoporous silica nanoparticles enable co-delivery of antigens and molecular adjuvants with precisely controlled release profiles. Magnetic nanoparticles combine vaccine delivery with imaging and magnetically guided targeting, whereas calcium phosphate nanoparticles offer excellent biocompatibility for biodegradable vaccine formulations. Carbon nanomaterials and nanozymes continue to expand opportunities for multifunctional hybrid immunotherapies [35,58].

Despite these advantages, clinical translation remains limited by several challenges. Long-term biopersistence, incomplete biodegradation, oxidative stress, potential accumulation within the liver and spleen, and uncertainties regarding chronic toxicity continue to raise regulatory concerns [164]. Furthermore, reproducible large-scale synthesis, rigorous control of physicochemical parameters, and standardization of manufacturing processes remain essential prerequisites for broader clinical implementation [165].

Current research increasingly focuses on hybrid nanoplatforms that combine biodegradable biological carriers with functional inorganic components. Such systems seek to preserve the unique optical, magnetic, and catalytic properties of inorganic materials while minimizing long-term toxicity through improved biodegradability and controlled elimination.

#### 2.4.6. Summary

Inorganic nanoplatforms represent multifunctional immunoengineering systems whose therapeutic potential extends well beyond conventional antigen delivery (Table 6). Their precisely engineered physicochemical properties enable integration of vaccine delivery, diagnostic imaging, externally controlled activation, and immune modulation within a single nanoscale architecture. Continued advances in surface engineering, biodegradable hybrid materials, catalytic nanotechnology, and externally responsive nanomaterials are expected to transform inorganic nanoparticles into key components of next-generation precision vaccines and personalized immunotherapies.

## 3. Future Perspectives of Nanoplatforms in Vaccinology

The rapid evolution of nanotechnology has fundamentally reshaped the conceptual framework of modern vaccinology. Initially developed as vehicles for protecting antigens and improving their delivery, nanoplatforms have progressively evolved into multifunctional immunoengineering systems capable of regulating virtually every stage of immune response. Contemporary vaccine nanotechnologies are no longer designed solely to enhance antigen stability or facilitate cellular uptake; rather, they integrate controlled antigen presentation, targeted delivery, intracellular trafficking, innate immune activation, and adaptive immune programming within a single nanoscale architecture. This convergence of materials science, molecular immunology, structural biology, and bioengineering is transforming vaccine development from an empirical process into a rational engineering discipline [166,167,168].

Unlike conventional vaccine formulations, in which the antigen and adjuvant represent largely independent components, next-generation nanoplatforms are increasingly conceived as integrated systems in which structural organization itself contributes to immunological function. Parameters such as particle geometry, mechanical properties, antigen valency, degradation kinetics, and surface chemistry are now recognized as programmable variables that collectively determine the magnitude, quality, and duration of vaccine-induced immunity [168,169]. Consequently, future vaccine design is expected to rely increasingly on predictive engineering principles that allow immune responses to be tailored according to specific pathogens, disease settings, and patient populations [170].

### 3.1. From Nanocarriers to Immune-Programming Platforms

Perhaps the most significant conceptual shift in nanovaccinology is the transition from passive antigen delivery toward active immune programming. Rather than serving merely as carriers that transport vaccine components to antigen-presenting cells, modern nanoplatforms are being engineered to influence multiple immunological processes simultaneously. By controlling lymphatic trafficking, cellular uptake pathways, intracellular antigen processing, and activation of innate immune signaling networks, nanoparticles can direct both the magnitude and polarization of adaptive immune responses [137,171].

This paradigm is particularly evident in platforms capable of coordinating humoral and cellular immunity within the same formulation. Through rational control of antigen organization, release kinetics, and adjuvant integration, nanoplatforms can promote efficient germinal center formation, enhance T follicular helper cell differentiation, improve cross-presentation through major histocompatibility complex class I pathways, and stimulate durable populations of memory B and T lymphocytes. Such coordinated immune programming is becoming increasingly important for vaccines targeting rapidly evolving viruses, chronic infectious diseases, intracellular pathogens, and malignant tumors, where robust antibody production alone is often insufficient to achieve long-term protection [172,173].

The concept of immune programming also extends beyond the induction of protective immunity. Emerging evidence indicates that nanoplatforms can be designed to modulate inflammatory responses, selectively activate distinct dendritic cell subsets, influence macrophage polarization, and regulate the local immune microenvironment. These capabilities position nanotechnology at the interface between vaccinology, immunotherapy, and precision medicine, expanding its applications from prophylactic vaccination toward therapeutic interventions for cancer, autoimmune diseases, and chronic inflammatory disorders [174].

### 3.2. Emerging Technological Directions

One of the most rapidly advancing areas in vaccine nanotechnology is the development of hybrid nanoplatforms that combine complementary properties of multiple delivery systems within a single multifunctional architecture. Hybrid nanoparticles integrating lipid, polymeric, protein, inorganic, or biomimetic components seek to overcome the limitations of individual platforms by simultaneously optimizing antigen stability, intracellular delivery, immune activation, and safety. For example, lipid–polymer hybrid nanoparticles combine the high encapsulation efficiency of lipid systems with the structural stability and controlled-release characteristics of biodegradable polymers, whereas biomimetic nanoparticles coated with natural cell membranes exploit endogenous biological recognition mechanisms to improve tissue targeting and immune compatibility. Similar strategies incorporating ferritin nanocages, virus-like particles, exosomes, or saponin-based immunostimulatory complexes are creating increasingly sophisticated vaccine platforms capable of integrating antigen delivery with immune modulation [175,176,177].

Another major direction is the development of stimuli-responsive nanomaterials capable of releasing antigens or activating immune responses only under predefined physiological or externally applied conditions. Such systems respond to local variations in pH, reactive oxygen species, enzymatic activity, temperature, or redox potential within infected tissues, tumors, or inflamed microenvironments. External physical stimuli—including light, ultrasound, magnetic fields, and electric fields—provide an additional level of spatial and temporal control, enabling precise activation of vaccine components at selected anatomical sites while minimizing systemic exposure. These programmable nanoplatforms may substantially improve therapeutic efficacy while reducing reactogenicity and off-target immune activation [178,179,180].

Parallel advances in structural biology and computational protein engineering are transforming the design of self-assembling vaccine nanoparticles. Artificial intelligence-assisted prediction of protein structure, molecular dynamics simulations, and de novo protein design now enable construction of nanoparticles with precisely controlled geometry, antigen orientation, and epitope spacing. These approaches facilitate the development of mosaic nanoparticles displaying antigens from multiple pathogen variants, thereby broadening immune recognition and supporting the design of universal vaccines against genetically diverse viruses such as influenza viruses, coronaviruses, and HIV [181,182,183].

Equally transformative is the emergence of precision vaccinology, which integrates genomic sequencing, immunopeptidomics, systems immunology, and computational modeling to tailor vaccine formulations according to individual immune profiles and disease characteristics. Personalized mRNA vaccines encoding patient-specific neoantigens have already demonstrated the feasibility of rapidly generating individualized therapeutic vaccines, particularly in oncology. Future nanoplatforms are expected to combine personalized antigen selection with programmable delivery systems capable of directing immune responses toward defined cellular targets, thereby improving therapeutic efficacy while minimizing adverse effects [184,185,186].

Artificial intelligence is expected to play an increasingly important role throughout the entire vaccine development pipeline. Beyond accelerating antigen discovery, machine-learning algorithms are being applied to optimize nanoparticle composition, predict physicochemical stability, model nanoparticle–cell interactions, identify correlates of protective immunity, and support adaptive clinical trial design. Integration of these computational approaches with high-throughput nanomaterial synthesis and systems vaccinology is likely to substantially reduce development timelines while improving the precision and reproducibility of vaccine design [187,188].

Collectively, these emerging technologies illustrate a broader transformation of nanovaccinology from material-centered platform development toward integrated immune engineering. Future vaccine systems are expected to function not merely as antigen carriers but as intelligent nanoscale devices capable of sensing biological environments, responding to external stimuli, and dynamically coordinating multiple components of innate and adaptive immunity. This evolution is anticipated to expand the therapeutic scope of nanoplatforms far beyond infectious disease prevention, establishing them as versatile technologies for cancer immunotherapy, treatment of chronic infections, and personalized precision medicine.

### 3.3. Clinical Translation: Opportunities and Remaining Challenges

The remarkable progress achieved in nanovaccine engineering over the past decade has accelerated the transition of several nanoplatforms from experimental systems to clinically validated technologies. Lipid nanoparticle-based mRNA vaccines have demonstrated that nanostructured delivery systems can be successfully implemented on a global scale, providing compelling proof-of-concept for the clinical feasibility of nanotechnology-enabled vaccination. Nevertheless, the translation of many emerging nanoplatforms—including polymeric nanoparticles, self-assembling protein nanostructures, exosomes, biomimetic vesicles, inorganic nanoparticles, and hybrid systems—continues to face substantial scientific, technological, manufacturing, and regulatory challenges. Addressing these barriers will be essential for expanding the clinical landscape of next-generation nanovaccines [38,189,190].

One of the principal obstacles remains large-scale manufacturing. Whereas laboratory-scale production allows precise control over nanoparticle composition and physicochemical characteristics, maintaining the same degree of uniformity during industrial manufacturing is considerably more complex. Minor variations in particle size distribution, antigen loading efficiency, surface functionalization, or encapsulation efficiency may significantly influence biodistribution, immunogenicity, and safety. Consequently, robust manufacturing processes capable of producing highly reproducible nanoparticle formulations under Good Manufacturing Practice (GMP) conditions have become a central objective of translational nanomedicine [191,192,193].

Microfluidic technologies have emerged as one of the most promising solutions for improving manufacturing consistency. Compared with conventional bulk mixing approaches, microfluidic systems enable highly controlled nanoparticle self-assembly by precisely regulating fluid dynamics, mixing rates, and solvent exchange. These technologies substantially reduce batch-to-batch variability, improve encapsulation efficiency, and facilitate continuous manufacturing processes that are readily scalable for industrial production. The successful implementation of microfluidic production during the rapid deployment of lipid nanoparticle-based mRNA vaccines has established an important technological framework for future nanovaccine manufacturing [194,195].

Beyond production itself, comprehensive physicochemical characterization remains a prerequisite for clinical development. Regulatory agencies increasingly require detailed evaluation of particle size distribution, morphology, surface charge, colloidal stability, antigen loading, release kinetics, and structural integrity throughout manufacturing, storage, and administration. Because many biological properties of nanoparticles are directly determined by these physicochemical parameters, standardized analytical methodologies and internationally harmonized quality-control criteria are becoming indispensable for regulatory approval [196,197].

Another major challenge concerns the regulatory classification of nanovaccines. Many contemporary platforms combine multiple functional components—including delivery systems, adjuvants, targeting ligands, and immunomodulators—within a single formulation, making them difficult to categorize within conventional regulatory frameworks. Depending on their composition and intended mechanism of action, nanovaccines may simultaneously exhibit characteristics of biological products, drug delivery systems, combination products, or advanced therapeutic medicinal products. Consequently, harmonization of regulatory requirements among international agencies will play an increasingly important role in facilitating global clinical implementation [198,199].

Clinical evaluation of nanovaccines also presents unique challenges. Traditional vaccine trials primarily assess safety, antibody titers, and protection against clinical disease. However, many modern nanoplatforms are specifically designed to modulate qualitative aspects of immunity, including T-cell polarization, germinal center dynamics, memory B-cell formation, mucosal immune responses, and innate immune training. Comprehensive evaluation of these mechanisms requires the incorporation of advanced immunological biomarkers, high-dimensional immune profiling, transcriptomic analyses, and systems vaccinology approaches capable of capturing the complexity of vaccine-induced immune responses. The integration of these technologies into clinical trial design is expected to improve both mechanistic understanding and prediction of long-term vaccine efficacy [200,201].

Long-term safety assessment represents another critical aspect of clinical translation. Although biodegradable lipid and polymeric nanoparticles generally demonstrate favorable safety profiles, important questions remain regarding repeated administration, tissue biodistribution, chronic exposure, and long-term biodegradation. Particular attention has been directed toward immune responses against polyethylene glycol (PEG), which may reduce delivery efficiency following repeated dosing and, in rare cases, contribute to hypersensitivity reactions. Likewise, inorganic nanomaterials require careful evaluation of long-term persistence, oxidative stress, and potential accumulation in biological tissues. Continued improvements in biodegradable materials, alternative surface coatings, and bioinspired nanostructures are expected to mitigate many of these concerns [31,202].

An additional consideration is the growing importance of personalized vaccinology, particularly in oncology and precision medicine. Advances in whole-genome sequencing, immunopeptidomics, and computational epitope prediction now enable rapid identification of patient-specific neoantigens that can be incorporated into individualized vaccine formulations. Lipid nanoparticle-mediated delivery of personalized mRNA vaccines has already demonstrated encouraging clinical activity in several cancers and illustrates the feasibility of producing customized vaccines within clinically relevant timeframes. Future developments are expected to integrate modular nanoplatforms with artificial intelligence-assisted antigen selection, thereby enabling increasingly individualized immunotherapeutic strategies while maintaining manufacturing scalability [203,204].

Economic and logistical considerations will also influence the future implementation of nanovaccines. The global deployment of mRNA vaccines during the COVID-19 pandemic highlighted both the strengths and limitations of current nanotechnology platforms. While rapid design and manufacturing represented a major technological achievement, stringent cold-chain requirements, complex production infrastructure, and unequal global distribution exposed vulnerabilities that remain to be addressed. Development of thermostable formulations, simplified manufacturing processes, and decentralized production technologies will therefore be essential for ensuring equitable access to future nanovaccines, particularly in low- and middle-income countries [205,206].

Taken together, these challenges emphasize that successful clinical translation requires a multidisciplinary approach extending far beyond nanoparticle engineering itself. Future progress will depend on the integration of advanced manufacturing technologies, standardized regulatory frameworks, comprehensive safety evaluation, systems immunology, computational modeling, and global public health infrastructure. Only through the coordinated development of these complementary disciplines can the full clinical potential of nanostructured vaccine platforms be realized (Table 7).

### 3.4. Outlook

The future of nanovaccinology will likely be shaped not by the discovery of entirely new nanomaterials, but by the convergence of multiple scientific disciplines into integrated vaccine engineering strategies. Advances in materials science, structural biology, synthetic biology, computational protein design, artificial intelligence, and systems immunology are progressively transforming nanoplatforms from passive delivery vehicles into intelligent immunoengineering systems capable of precisely controlling the magnitude, quality, and duration of immune responses.

A major trend is the increasing modularity of nanovaccine design. Rather than relying on a single carrier technology, future platforms are expected to combine complementary structural and functional elements—including programmable antigen display, targeted delivery, controlled intracellular trafficking, and integrated immunostimulatory signaling—within unified nanoscale architectures. Such modular systems will facilitate rapid adaptation to emerging pathogens, newly identified tumor antigens, and evolving clinical requirements while maintaining manufacturing flexibility and regulatory compatibility.

The growing application of computational modeling and artificial intelligence is expected to further accelerate vaccine development by enabling predictive optimization of nanoparticle composition, antigen organization, and immune responses before experimental validation. Integration of high-throughput materials screening with machine learning, digital manufacturing, and systems vaccinology may substantially shorten development timelines while improving reproducibility and translational success.

Future nanoplatforms are also likely to expand beyond conventional prophylactic vaccination. The same engineering principles that govern antigen delivery and immune programming may be applied to therapeutic cancer vaccines, chronic viral infections, antimicrobial resistance, autoimmune disorders, allergy immunotherapy, and even regenerative medicine. As nanotechnology increasingly interfaces with precision medicine, vaccine platforms may evolve toward individualized therapeutic systems capable of adapting to patient-specific immunological profiles and dynamic disease progression.

Ultimately, the next generation of nanovaccines will be defined not by the materials from which they are constructed, but by their capacity to integrate sensing, targeting, immune modulation, and therapeutic intervention within programmable nanoscale architectures. Achieving this vision will require continued collaboration among immunologists, materials scientists, engineers, clinicians, computational biologists, regulatory agencies, and manufacturers. Such interdisciplinary integration is expected to establish nanotechnology as one of the fundamental enabling technologies of precision immunomedicine.

## 4. Conclusions

Nanotechnology has fundamentally transformed modern vaccinology by providing versatile platforms capable of integrating antigen delivery, immune modulation, and controlled activation of adaptive immunity within rationally engineered nanoscale systems. The diverse nanoplatforms discussed in this review—including lipid and polymeric nanoparticles, nanoemulsions, biomimetic vesicles, saponin-based immunostimulatory complexes, self-assembling protein nanoparticles, and inorganic nanomaterials—demonstrate that vaccine performance is increasingly determined by the engineering of physicochemical architecture rather than by antigen formulation alone.

Despite substantial differences in material composition and biological origin, these platforms converge on a common set of engineering principles. Structural organization governs antigen presentation and multivalency; particle size, surface chemistry, and mechanical properties regulate biodistribution and cellular trafficking; while controlled activation of innate immune pathways shapes the magnitude and polarization of adaptive immune responses. Collectively, these interconnected mechanisms provide a unified conceptual framework for understanding how nanostructured vaccines generate protective immunity.

The successful clinical implementation of lipid nanoparticle-based mRNA vaccines has demonstrated the transformative potential of nanotechnology for global vaccination programs and has accelerated the development of numerous next-generation vaccine platforms. At the same time, continued progress will require solutions to several outstanding challenges, including scalable manufacturing, standardized physicochemical characterization, harmonized regulatory pathways, comprehensive long-term safety assessment, and equitable global access. Addressing these issues will be essential for translating promising experimental nanoplatforms into broadly applicable clinical technologies.

Looking forward, nanovaccinology is expected to progress toward increasingly intelligent, multifunctional, and personalized vaccine systems. The integration of advanced materials engineering, synthetic biology, computational protein design, artificial intelligence, and systems immunology will enable the development of programmable nanoplatforms capable of directing immune responses with unprecedented spatial, temporal, and molecular precision. Rather than functioning solely as delivery vehicles, future nanovaccines are likely to become adaptive immunoengineering platforms that coordinate antigen presentation, immune sensing, and therapeutic modulation within a single nanoscale architecture.

**Figure 2:**
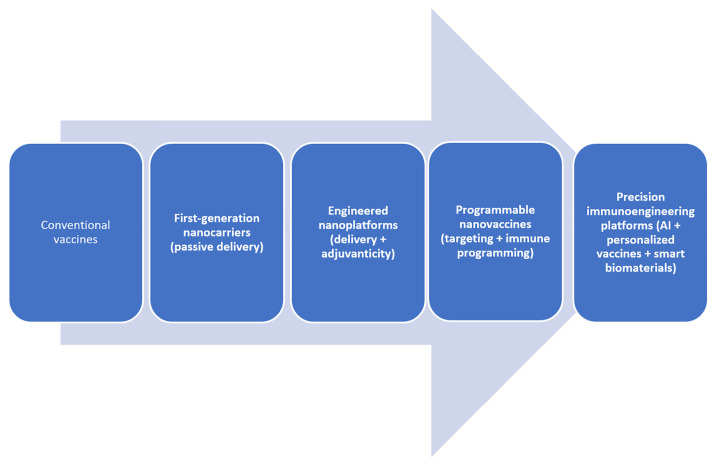
Evolution of nanoplatforms in modern vaccinology.

In conclusion, the continuing convergence of nanotechnology and immunology is redefining the principles of vaccine design. By combining structural precision with functional adaptability, nanoplatforms provide a versatile foundation for the development of next-generation prophylactic and therapeutic vaccines against infectious diseases, cancer, and other immune-mediated disorders (Figure 2, Table 8).

As manufacturing technologies, regulatory science, and systems immunology continue to advance, nanotechnology is poised to become a central pillar of precision vaccinology and one of the key drivers of future immunotherapeutic innovation.

## Figures and Tables

**Table 1:** Physicochemical design parameters governing nanoparticle vaccine performance.

Design Parameter	Primary Biological Effect	Immunological Consequence	References
Particle size	Lymphatic drainage, tissue diffusion	APC targeting, CD8^+^ T-cell priming, germinal center formation	[13,14]
Surface charge (ζ-potential)	Membrane interaction, protein adsorption	Cellular uptake, reactogenicity	[15,16]
Surface chemistry	Protein corona formation	Biodistribution, complement activation	[16,17]
Particle shape	Endocytosis and circulation behavior	APC uptake efficiency	[18,19]
Elasticity (stiffness)	Cellular deformation and internalization	Intracellular trafficking	[20,21]
Antigen density	B-cell receptor crosslinking	Humoral immunity, affinity maturation	[22,23,24]
Antigen localization	Antigen processing pathway	MHC-I/MHC-II presentation	[25,26]
Degradation kinetics	Duration of antigen exposure	Immune persistence and memory	[26]
Endosomal escape	Cytosolic antigen delivery	Cross-presentation and CTL activation	[27,28]
Targeting ligands	Cell-specific recognition	Tissue-selective immune responses	[29,30]
PEGylation	Extended circulation	Increased stability but potential anti-PEG immunity	[31]

**Table 2:** Categories of nanovaccine systems.

Nanoplatform Category	Representative Systems	Main Characteristics	Advantages in Vaccine Delivery	Limitations/ Challenges	References
Lipid-, polymer-, and hybrid nanoparticle systems	lipid nanoparticles (LNPs), liposomes, solid lipid nanoparticles (SLNs), nanoemulsions, polymeric systems based on poly(lactic-co-glycolic acid) (PLGA), polylactic acid/polyglycolic acid (PLA/PGA), hybrid lipid-polymer systems solid lipid nanoparticles (SLNs), chitosan-derived nanocarriers and copolymers	Biodegradable colloidal carriers capable of encapsulating proteins and nucleic acids with controlled release and surface functionalization	Efficient delivery of mRNA/DNA and protein antigens, protection from degradation, sustained release, targeted delivery, endosomal escape	Limited storage stability, manufacturing complexity, potential reactogenicity of some formulations	[38,39,40]
Self-Assembling Protein Nanoplatforms: Virus-Like Particles and Ferritin Nanocages	Virus-like particles (VLPs), ferritin nanocages	Self-assembling protein nanostructures lacking genetic material that mimic viral morphology	Highly repetitive antigen display, strong B-cell activation, robust humoral immunity, excellent safety, multivalent antigen presentation	Restricted cargo loading, complex antigen engineering, relatively high production costs	[41,42,43]
Saponin-Based Self-Assembling Nanoplatforms	ISCOMs, ISCOMATRIX^®^, Matrix-M	Cage-like nanoparticles composed of saponins, cholesterol, and phospholipids acting primarily as adjuvant systems	Potent induction of humoral and cellular immunity, cross-presentation, balanced Th1/Th2 responses, mucosal immunity	Saponin-associated reactogenicity, formulation complexity, limited number of licensed products	[44,45,46,47,48]
Inorganic Nanoplatforms for Vaccine Delivery and Immunotherapy	Gold nanoparticles, silica nanoparticles, iron oxide nanoparticles, aluminum nanostructures, carbon nanotubes, graphene derivatives	Structurally stable inorganic nanomaterials with unique optical, magnetic, and physicochemical properties	High antigen loading, surface modification imaging, magnetic targeting, theranostic and multifunctional applications	Limited biodegradability, uncertain long-term toxicity, biodistribution and regulatory concerns	[49,50,51,52,53,54,55,56,57]

**Table 3:** Mechanistic comparison of major nanoparticle platforms used in vaccine delivery.

Characteristic	Lipid Nanoparticles (LNPs)	Polymeric Nanoparticles	Nanoemulsions	Extracellular Vesicles/Biomimetic Vesicles
Typical composition	Ionizable lipids, cholesterol, phospholipids, PEG-lipids	PLGA, PLA, PGA, chitosan, PCL	Oil droplets stabilized by surfactants	Natural lipid bilayers with membrane proteins and nucleic acids
Primary cargo	mRNA, siRNA, DNA	Proteins, peptides, DNA, RNA, adjuvants	Protein antigens, hydrophobic antigens, adjuvants	Proteins, RNA, peptides, small molecules
Antigen loading strategy	Encapsulation	Encapsulation or surface adsorption	Interfacial adsorption	Natural incorporation or engineered loading
Controlled release	Limited (transient expression)	Excellent	Moderate (depot effect)	Moderate
Endosomal escape	Excellent	Low–moderate (requires engineering)	Minimal	Moderate
Lymphatic drainage	High	High	Moderate	Moderate–high
Targeting capability	Passive; ligand modification possible	Passive; easily functionalized	Mostly passive	Intrinsic receptor-mediated targeting
Innate immune activation	Moderate–high	Moderate	High	Low (engineerable)
Cross-presentation (MHC-I)	Excellent	Moderate	Low	Moderate–high
Humoral immunity	High	High	Very high	High
Cellular immunity	Excellent	High	Moderate	High
Intrinsic adjuvanticity	Moderate	Moderate	Strong	Weak
Protein corona formation	Pronounced	Pronounced	Moderate	Minimal
Complement activation	Possible (CARPA risk)	Low	Low	Minimal
Biocompatibility	High	High	High	Very high
Manufacturing scalability	Excellent	Good	Excellent	Limited
Batch reproducibility	High	Moderate	High	Low
Clinical maturity	Very high	Moderate	High	Emerging
Representative applications	mRNA vaccines, gene delivery	Controlled-release vaccines, cancer vaccines	Adjuvant formulations, mucosal vaccines	Precision immunotherapy, personalized vaccines

**Table 4:** Immunological characteristics of major nanoparticle vaccine platforms.

Platform	APC Uptake	Endosomal Escape	Cross- Presentation	Germinal Center Induction	Mucosal Delivery	Reactogenicity
LNP	★★★★★	★★★★★	★★★★★	★★★★☆	★★☆☆☆	★★★★☆
Polymeric NP	★★★★☆	★★☆☆☆	★★★☆☆	★★★★★	★★★★☆	★★☆☆☆
Nanoemulsion	★★★☆☆	★☆☆☆☆	★★☆☆☆	★★★★★	★★★★★	★★★★☆
Extracellular vesicles	★★★★★	★★★☆☆	★★★★☆	★★★★☆	★★★☆☆	★☆☆☆☆

The maximum activity score is presented on a 5-point scale (stars); black stars indicate the score.

**Table 5:** Comparison of self-assembling protein nanoplatforms.

Characteristic	Virus-like Particles	Ferritin Nanocages
Structural origin	Viral structural proteins	Endogenous ferritin
Self-assembly	Viral capsid assembly	24-subunit nanocage
Typical size	20–200 nm	~12 nm
Antigen display	Native viral epitopes	Engineered antigen fusion/conjugation
Intrinsic adjuvanticity	High	Low–moderate
Cross-presentation	Efficient	Moderate–high
Humoral immunity	Excellent	Excellent
Cellular immunity	High	High (with adjuvants)
Engineering flexibility	Moderate	Very high
Clinical maturity	Licensed vaccines	Clinical trials

**Table 6:** Functional characteristics of major inorganic nanoparticle platforms in vaccinology.

Nanoplatform	Unique Physicochemical Property	Primary Vaccine Function	Advantages	Major Limitations
Gold nanoparticles (AuNPs)	Surface plasmon resonance	Multivalent antigen display, photothermal immunotherapy	High stability, easy functionalization	Poor biodegradability
Silver nanoparticles (AgNPs)	Antimicrobial activity	Antigen delivery, antimicrobial vaccination	Intrinsic antimicrobial effects	Cytotoxicity, oxidative stress
Mesoporous silica nanoparticles (MSNs)	High porosity	Antigen and adjuvant encapsulation	Large loading capacity, controlled release	Slow degradation
Iron oxide nanoparticles	Magnetism	Magnetic targeting, MRI, hyperthermia	Image-guided delivery, external control	Long-term accumulation
Calcium phosphate nanoparticles	Biodegradability	Vaccine carrier	Excellent biocompatibility	Lower structural versatility
Carbon nanomaterials	High surface area, electrical conductivity	Multifunctional delivery platforms	Extensive functionalization	Variable biocompatibility
Quantum dots	Fluorescence	Imaging and antigen tracking	Real-time visualization	Heavy-metal toxicity concerns
Nanozymes	Enzyme-like catalytic activity	Immune regulation, ROS modulation	Programmable catalytic function	Early stage of development

**Table 7:** Major challenges and emerging solutions for the clinical translation of nanovaccines.

Challenge	Current Limitations	Emerging Solutions
Manufacturing	Batch-to-batch variability; scale-up difficulties	Microfluidic and continuous manufacturing; automated process control
Physicochemical characterization	Lack of standardized analytical methods	International harmonization of quality-control protocols
Regulatory approval	Complex classification of multifunctional nanoplatforms	Nanotechnology-specific regulatory guidance and standardized evaluation criteria
Clinical evaluation	Limited correlates of protection beyond antibody responses	Systems vaccinology, immune profiling, multi-omics biomarkers
Long-term safety	Biodistribution, repeated dosing, biodegradation, anti-PEG immunity	Biodegradable materials, alternative surface chemistries, long-term pharmacovigilance
Personalized vaccination	Time-consuming individualized manufacturing	Modular nanoparticle platforms integrated with AI-assisted antigen selection
Global accessibility	Cold-chain dependence and high production costs	Thermostable formulations, decentralized and scalable manufacturing

**Table 8:** Major challenges and future directions for clinical translation of nanovaccines.

Challenge	Current Limitation	Emerging Solutions
Manufacturing	Batch variability	Microfluidics, continuous manufacturing
Safety	Long-term biodistribution	Biodegradable materials
Regulatory approval	Lack of harmonized guidelines	Standardized characterization
Personalized vaccines	Manufacturing time	AI-assisted antigen design, modular LNP platforms
Stability	Cold-chain dependence	Thermostable formulations
Clinical monitoring	Limited biomarkers	Systems vaccinology and immune profiling

## Data Availability

All the data generated or analyzed during this study have been included in the published article.
